# Identification of Novel Biomarkers With Diagnostic Value and Immune Infiltration in Burn Injury

**DOI:** 10.3389/fgene.2022.829841

**Published:** 2022-03-22

**Authors:** Sitong Zhou, Kangchun Wang, Jingru Wang, Jia He, Wenlian Zheng, Chengmin Long, Xiaodong Chen, Ronghua Yang

**Affiliations:** ^1^ Department of Dermatology, the First People’s Hospital of Foshan, Foshan, China; ^2^ Department of Organ Transplantation and Hepatobiliary, the First Affiliated Hospital of China Medical University, Shenyang, China; ^3^ Department of Burn Surgery and Skin Regeneration, the First People’s Hospital of Foshan, Foshan, China; ^4^ Graduate School, Guangdong Medical University, Zhanjiang, China; ^5^ Department of Burn and Plastic Surgery, Guangzhou First People’s Hospital, School of Medicine, South China University of Technology, Guangzhou, China

**Keywords:** burn injury, inflammation, immune infiltration, functional enrichment analysis, CIBERSORT

## Abstract

Burn injury is an intractable problem in the field of surgery where screening relevant target genes and exploring pathological mechanisms through bioinformatic methods has become a necessity. Herein, we integrated three burn injury mRNA microarray datasets from the Gene Expression Omnibus database to analyze the hub differentially expressed genes (DEGs) between burn injury patient samples and healthy human samples; we conducted multiple functional enrichment analyses and constructed the protein–protein interaction (PPI) network. Finally, we evaluated the immune infiltration in the burn injury microenvironment. A total of 84 intersection DEGs (32 upregulated and 52 downregulated) were screened in burn injury patients *via* integrated analyses. Upregulated genes were primarily enriched in regulation of T cell activation, regulation of response to DNA damage stimulus, positive regulation of innate immune response, positive regulation of defense response. We also identified 10 hub genes from the PPI network (CCNB2, MYO10, TTK, POLQ, VASP, TIMP1, CDK16, MMP1, ZYX, and PKMYT1). Next, we found that 22 immune cells were substantially changed during the burn injury by CIBERSORT. In addition, we verified that VASP and POLQ are two novel diagnostic markers in burn processes with high diagnostic efficacy *via* immunohistochemistry. In summary, we identified several key genes involved in burn injury and provided a favorable basis for elucidating the molecular mechanisms of burn injury through comprehensive bioinformatic analysis.

## Introduction

Burn injury primarily refers to damage to tissues, including skin and mucous membranes, caused by heat. In some severe cases, it can also involve subcutaneous and submucosal tissues and even internal organs. According to statistics from the World Health Organization, approximately 300,000 people die from burn injuries worldwide every year (Roshangar et al., 2019). In particular, the healing of a deep burn injury is a complex process involving multiple factors and an intractable problem in the surgical field (Gerber et al., 2019). Burn injury not only damages the cells, tissues, and blood vessels but also affects the release of various growth factors and cytokines. Severe burns may be accompanied by insufficient blood supply, severe infections, and even sepsis, which will seriously prolong the wound healing time. In addition, a burn injury usually requires long-term treatment and multiple reconstructive surgical operations, which may cause irreversible damage to the patient’s mental health. Studies have shown that burn patients often show a tendency of depression and require long-term psychological treatment, which seriously affects their life quality (Smolle et al., 2017).

Currently, bioinformatic analysis is an important tool for analyzing the expression data and screening for target genes in many diseases. The full use of gene detection technology and bioinformatics will effectively explore the mechanism of various diseases, including burn injury. Numerous studies have revealed that there are many gene expression changes at different stages of burn injury (Fang et al., 2020). A strong immune and stress response disorder is one of the most important features in the early stage of burn injury, and many studies indicate that the high-mobility group box protein 1, a nuclear protein, is increased in burn injury patients, which is due to it passive release from damaged cells (Lantos et al., 2010). In the repair stage, various molecules are involved in scar formation. Transforming growth factor β1 (TGF-β1) can mediate fibrosis of wounds and form hypertrophic scars after burning; Smad7 can negatively regulate the TGF-β1/Smad pathway, thereby preventing fibrosis mediated by TGF-β1 (Zhang et al., 2020). Molecular changes can also be observed in some serious burn complications. Cystic fibrosis transmembrane conductance regulator and downstream signaling were critical in modulating the gut ischemia and hypoxia post severe burn, which resulted in sepsis and multiple organ failure (Liu et al., 2020).

Most burn injury–related studies use single microarrays or mouse microarrays (Gao et al., 2016; Zou et al., 2017). However, key differentially expressed genes (DEGs) and biological pathways identified in these burn injury studies present a high false-positive rate in a single microarray and a low level of evidence for mouse microarrays, limiting the accuracy of the results. Further comprehensive analysis is needed to identify the key molecular markers and diagnostic targets. This study integrates three burn injury mRNA microarray datasets from the Gene Expression Omnibus (GEO) database to analyze the hub DEGs between burn injury patients and healthy human samples. We conducted multiple functional enrichment analyses and constructed a protein–protein interaction (PPI) network; finally, we explored the immune infiltration in the burn injury microenvironment and we verified that VASP and POLQ are two novel diagnostic markers in burn processes with high diagnostic efficacy via immunohistochemistry. Our study aimed to identify several key genes involved in burn injury and provide a favorable basis for elucidating its underlying molecular mechanisms.

## Materials and Methods

### Data Source and Differential Expression Analysis

We downloaded three microarray datasets of burn injury tissue and normal tissue from the GEO dataset^1^ for further analysis (GSE8056, GSE19743, GSE37069) (Barrett et al., 2013). Next, we performed principal component analysis (PCA) and differential expression analysis (Ringnér, 2008). We divided the data into two groups: the burn injury group and the normal group and used the R package “limma” to analyze datasets and screen out DEGs (Ritchie et al., 2015). Thresholds of |log2FC| > 1.0 and an adjusted *p*-value < 0.05 were selected.

### Functional Enrichment Analysis

Metascape^2^ is a powerful online tool for analyzing gene function annotation (Zhou et al., 2019) and provides gene enrichment analysis and PPI network analyses. This tool was used to analyze the gene ontology (GO) and the Kyoto Encyclopedia of Genes and Genomes (KEGG) pathway enrichment analysis. In addition, a series of analyses were performed in the DisGeNET database (Piñero et al., 2017), Trrust database (Han et al., 2018), and PaGenbase database (Pan et al., 2013).

### Construction of PPI Network

The STRING database^3^ is a practical online tool that can be used to construct PPI networks (Szklarczyk et al., 2015). Since the upregulated DEGs were highly enriched in functions and pathways that are closely related to burn injury and immune response, all the upregulated DEGs were included in the database for analysis, and the interaction threshold was set to 0.4. The Cytoscape software was used for visualization (Doncheva et al., 2019), and CytoHubba was used to analyze hub genes in the network (Chin et al., 2014). In the PPI network, we screened 10 hub genes, and correlation was shown by different shades of colors.

### Gene Set Enrichment Analysis (GSEA)

To identify the signal transduction pathways that were differentially activated between the burn injury group and the normal group, we selected an ordered list of genes through the “limma” R package and then performed a gene set enrichment analysis.

### Expression Level and Receiver Operating Characteristic (ROC) Curves of Top 10 Hub Genes.

We analyzed the expression levels of 10 hub genes in burn injury and normal tissues in the GSE37069 dataset and constructed a columnar scatter plot. In addition, using the package “pROC” to perform the ROC analysis (Robin et al., 2011), the specificity and sensitivity of each gene were obtained, and the area under the curve (AUC) of each hub gene was calculated.

### CIBERSORT

CIBERSORT^4^ can enumerate cell type composition in gene expression data through deconvolution algorithm, and it has been used to evaluate immune cell infiltration in many diseases, estimated abundances of immunocytes had been assessed by 22 given kinds of immunocytes accompanying with 1,000 permutations. Then, the immune cell matrix was visualized by the R package of “ggplot2”. Finally, we constructed the correlation heatmap for visualizing the correlation of infiltrating immune cells by “corrplot” package.

### Patient Tissue Specimens

Fifteen clinical samples were obtained from the Chinese Han population from 2020 to 2021. This study was approved by the Ethics Committee of Foshan First People’s Hospital (AF-SOP-20-1.4-0). All subjects provided written informed consent, in accordance with the Declaration of Helsinki.

### Immunohistochemistry (IHC) Staining and Histologic Scoring

Paraffin-embedded tissues were sectioned at 4 μm for IHC analysis. Antigen retrieval was performed by incubating the samples in citrate buffer (pH 6.0) for 15 min. After blocking with a mixture of methanol and 0.75% hydrogen peroxide, sections were incubated overnight with primary antibody (VASP, Signalway Antibody, 1:100; POLQ, Signalway Antibody, 1:50), followed by incubation with a secondary antibody conjugated with horseradish peroxidase (goat anti-rabbit, 1:500, Cell Signaling Technology). Sections were washed three times with phosphate-buffered saline and incubated with diaminobenzidine. The process of histologic scoring and analysis was as described in our previous study (Zhou et al., 2021).

## Results

### Differential Expression Analysis Results

**FIGURE 1:**
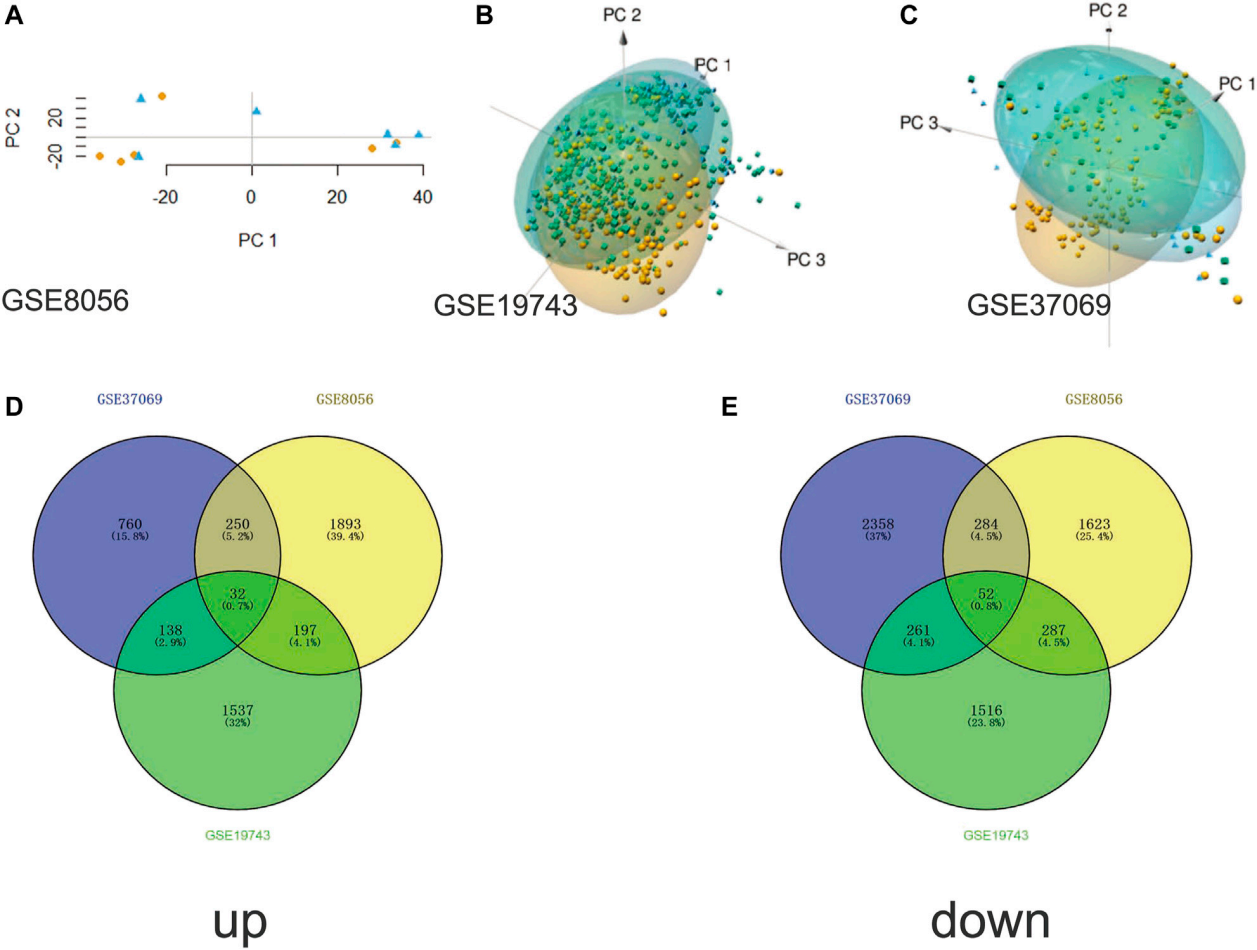
Principal component analysis and Venn plots of DEGs. **(A)** PCA results of GSE8056 dataset; **(B)** PCA results of GSE19743 dataset; **(C)** PCA results of GSE37069 dataset; **(D)** Venn plot of upregulated DEGs; **(E)** Venn plot of downregulated DEGs.

**FIGURE 2:**
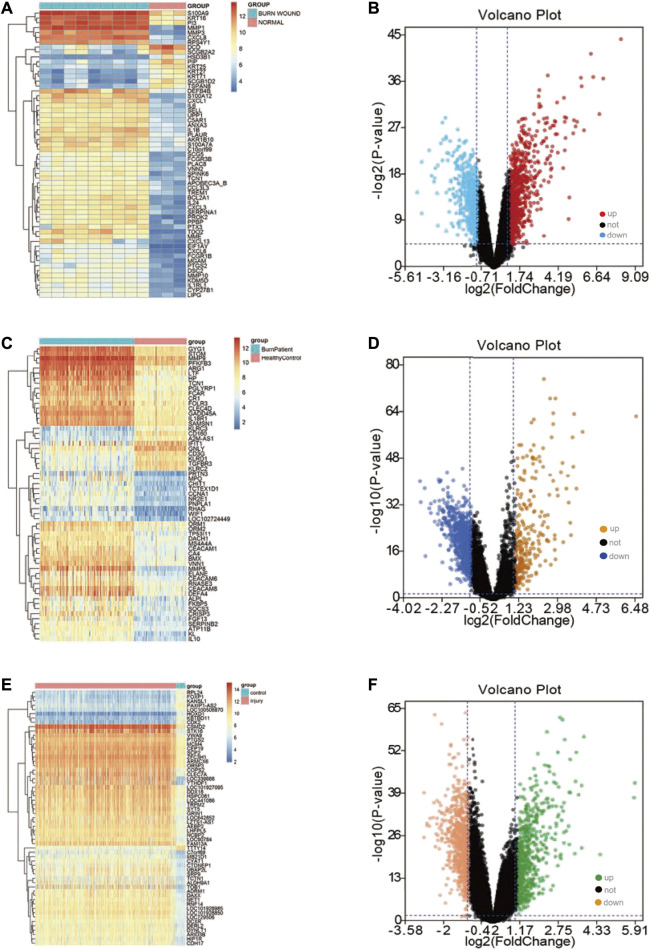
Differential expression analysis in three datasets. **(A)** The gene expression heatmap of important DEGs in the GSE8056 dataset; **(B)** the volcano plot of the differential expression analysis in the GSE8056 dataset; **(C)** the gene expression heatmap of important DEGs in the GSE19743 dataset; **(D)** volcano plot of differential expression analysis in GSE19743 dataset; **(E)** the gene expression heatmap of important DEGs in the GSE37069 dataset; **(F)** volcano plot of differential expression analysis in GSE37069 dataset.

We obtained three datasets (GSE8056, GSE19743, GSE37069) from the GEO database, including 779 samples (676 burn patients and 103 healthy controls). PCA revealed that burn patients and healthy control samples in the three datasets showed a significantly different gene expression profile (Figures 1A–C). Next, we separately conducted a differential expression analysis of the three datasets and analyzed their intersection. Finally, 32 upregulated and 52 downregulated DEGs were obtained (Figures 1D,E). In addition, volcano maps of DEGs and heatmaps of important DEGs are displayed in Figure 2.

### Functional Enrichment Analysis

**FIGURE 3:**
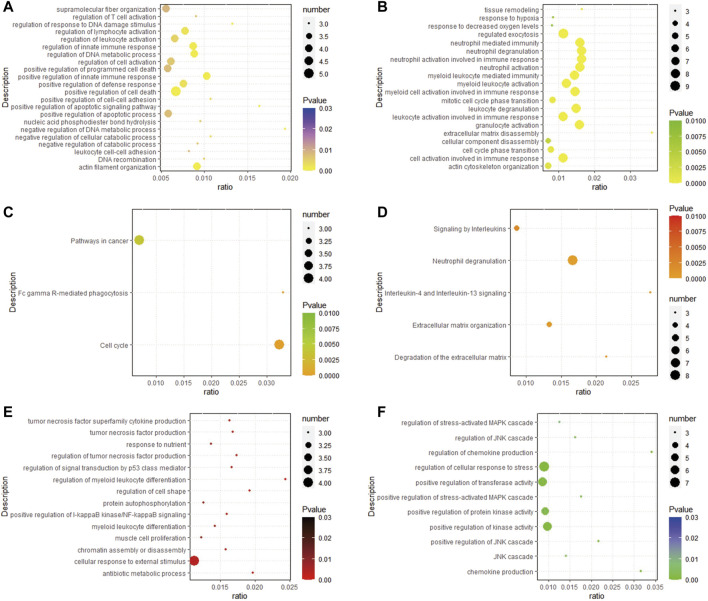
Analysis results of functional enrichment. **(A,B)** GO analysis of up-regulated DEGs; **(C)** enrichment analysis of the KEGG of up-regulated DEGs; **(D)** enrichment analysis of the REACTOME pathway of up-regulated DEGs; **(E,F)** GO analysis of down-regulated DEGs.

According to the results of GO, the upregulated DEGs were primarily enriched in regulation of T cell activation, regulation of response to DNA damage stimulus, positive regulation of innate immune response, positive regulation of defense response, positive regulation of cell-cell adhesion, positive regulation of apoptotic signaling pathway, neutrophil activation involved in immune response, myeloid cell activation involved in immune response, mitotic cell cycle phase transition, leukocyte degranulation, leukocyte activation involved in immune response, extracellular matrix disassembly, cellular component disassembly (Figures 3A,B). In addition, the upregulated DEGs were primarily enriched in pathways in cancer, Fc gamma R-mediated phagocytosis, and cell cycle (Figure 3C). In the Reactome database, neutrophil degranulation, signaling by interleukins and extracellular matrix organization are some important pathways (Figure 3D). The GO of downregulated DEGs were primarily enriched in tumor necrosis factor production, response to nutrient, cellular response to external stimulus, regulation of chemokine production, regulation of cellular response to stress, positive regulation of transferase activity, positive regulation of protein kinase activity (Figures 3E,F).

**FIGURE 4:**
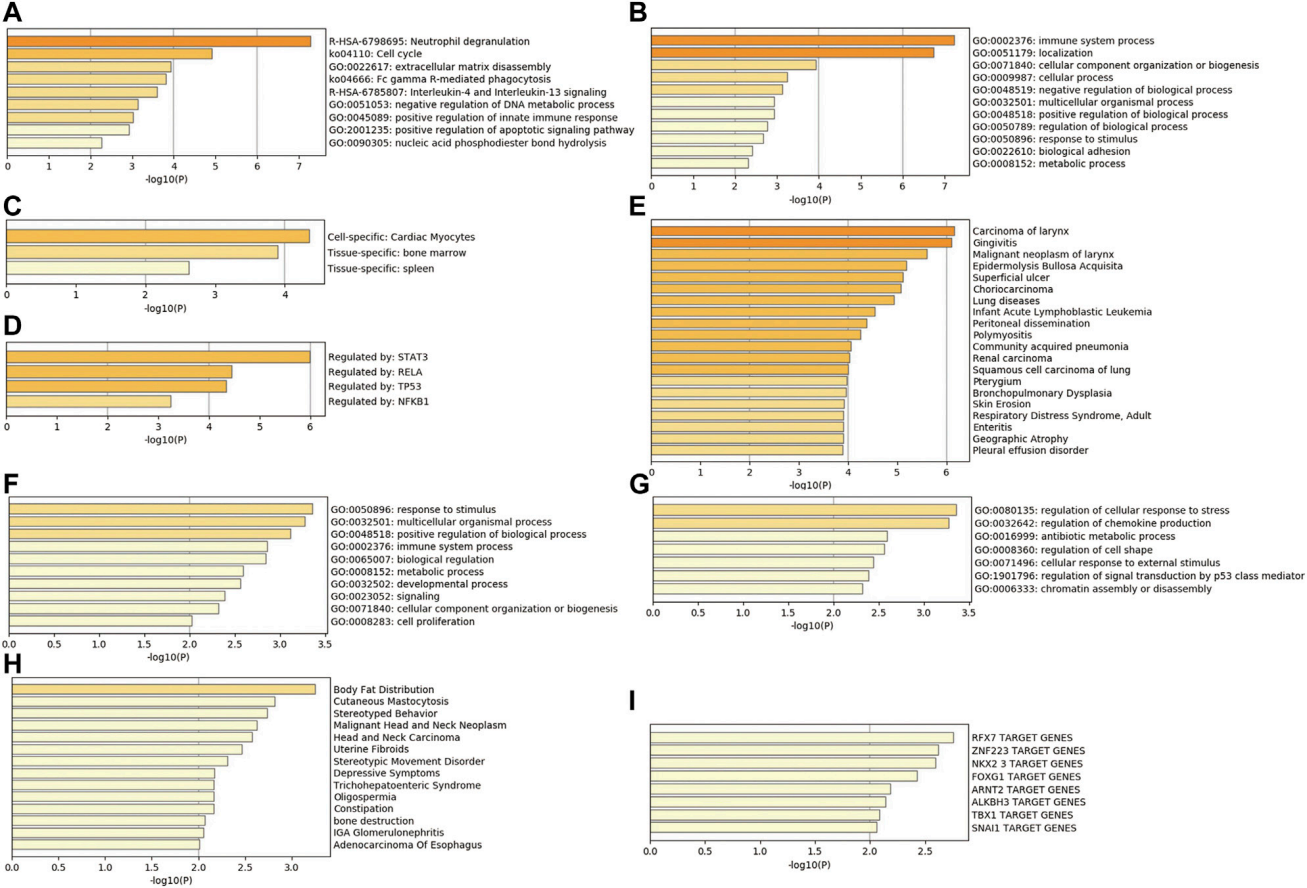
Comprehensive database analysis. **(A,B)** Biological functions of upregulated genes analyzed through Metascape database; **(C)** tissue and cell characteristics of upregulated genes in PaGenBase database; **(D)** enrichment of transcriptional regulators of upregulated genes in TRRUST database; **(E)** DisGeNET database enrichment analysis of diseases involving upregulated genes; **(F,G)** Biological functions of downregulated genes analyzed through Metascape database; **(H)** DisGeNET database enrichment analysis of diseases involving downregulated genes; **(I)** enrichment of transcriptional regulators of downregulated genes in TRRUST database.

In addition, the analysis results of a series of databases are shown in Figure 4, PaGenBase database analysis showed that upregulated genes were mainly enriched in the cardiac myocytes (cell-specific), bone marrow, and spleen (tissue-specific). TRRUST database analysis uncovered STAT3, RELA, TP53, and NF-κB1 to be the main transcription factors regulating the upregulated genes. Besides, DisGeNET database disease enrichment analysis demonstrated that the upregulated genes were associated with carcinoma of larynx, gingivitis, malignant neoplasm of larynx. Compared with the downregulated DEGs, the upregulated genes were more involved in immune-related functions, which may be closely related to the stress and immune responses triggered by the burn injury.

### PPI Network and Hub Genes

**FIGURE 5:**
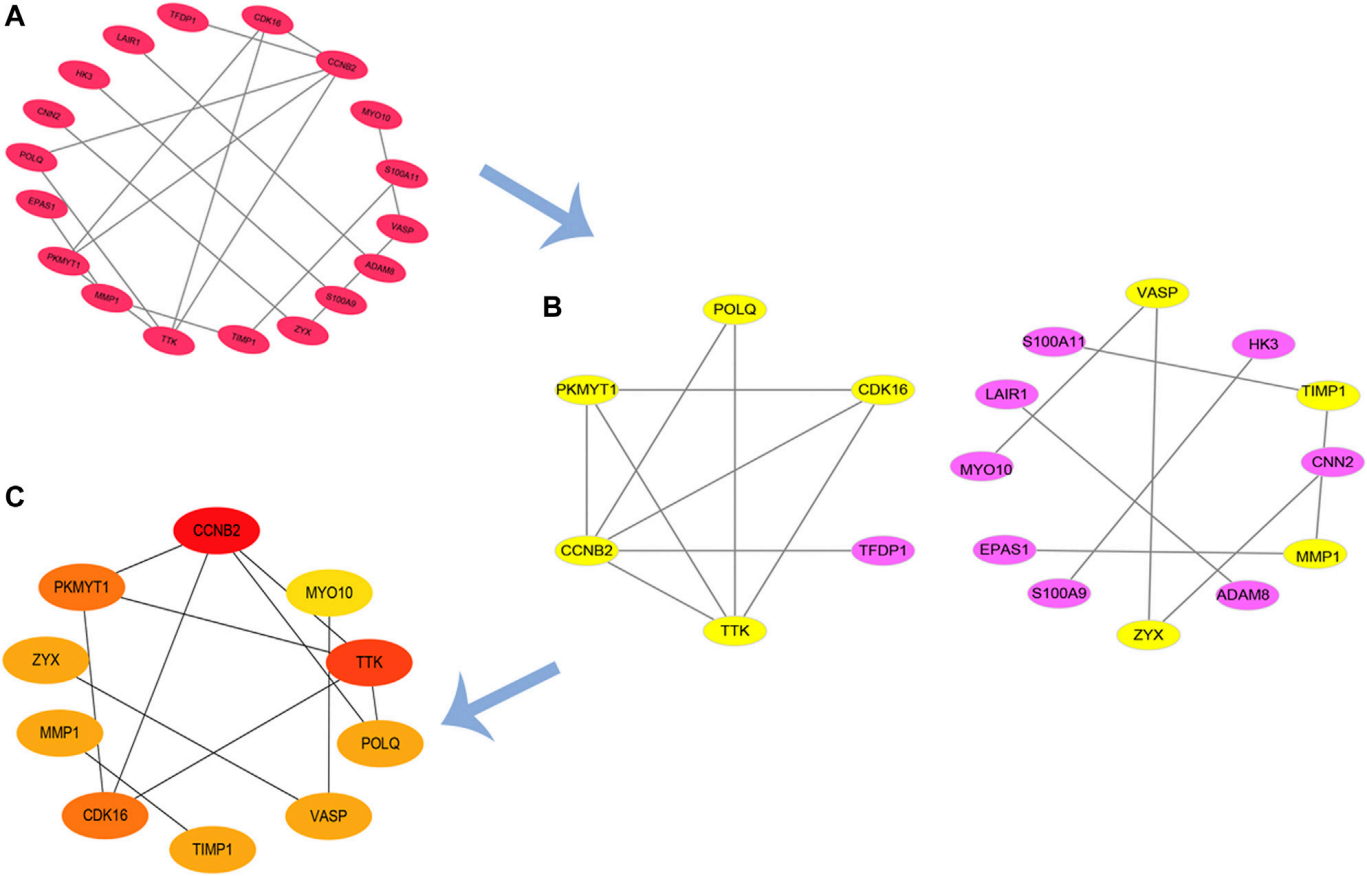
PPI network of upregulated DEGs and hub genes. **(A,B)** PPI network of up-regulation DEGs; **(C)** identified hub genes by CytoHubba.

Based on our previous findings, we constructed a PPI network of upregulated genes by Cytoscape software and we analyzed this network (Figures 5A,B). Finally, using the CytoHubba to analyze hub genes, we identified 10 genes with the highest scores (CCNB2, MYO10, TTK, POLQ, VASP, TIMP1, CDK16, MMP1, ZYX, and PKMYT1) (Figure 5C). We suggest that these 10 genes are hub genes in the PPI network, and play an important role in the basic pathological process of burn injury, with the potential to serve as important markers in burn injury.

### GSEA

**FIGURE 6:**
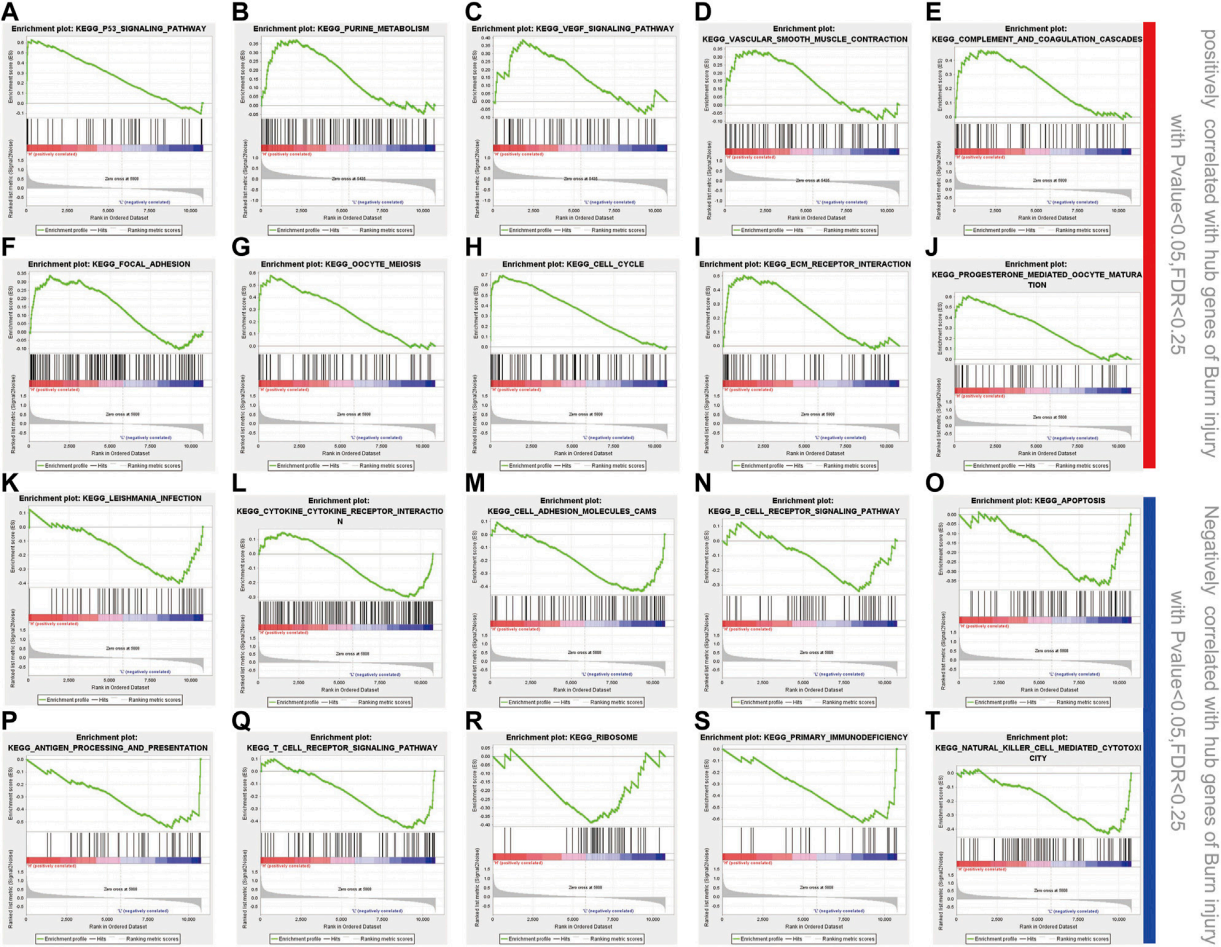
GSEA enrichment analysis. **(A-J)** 10 positively correlated pathways in burn injury; **(K-T)** 10 negatively correlated pathways in burn injury.

Twenty important KEGG pathways were identified in the GSEA, including 10 pathways that were positively related to key genes, and 10 pathways that were negatively related key genes, including the following important pathways: P53 signaling pathway, purine metabolism, VEGF signaling pathway, vascular smooth muscle contraction, complement and coagulation cascades, focal adhesion, oocyte meiosis, cell cycle, ECM receptor interaction, progesterone mediated oocyte maturation (positively correlated) (Figures 6A–J). Leishmania infection, cytokine-cytokine receptor interaction, cell adhesion molecules (CAMs), B cell receptor signaling pathway, apoptosis, antigen processing and presentation, T cell receptor signaling pathway, ribosome, primary immunodeficiency, natural killer cell mediated cytotoxicity (negatively correlated) (Figure 6K-T). These results suggest that hub genes may be involved in these signaling pathways and have important effects on burn injury.

### Expression Level and ROC Curves of Top 10 Hub Genes

**FIGURE 7:**
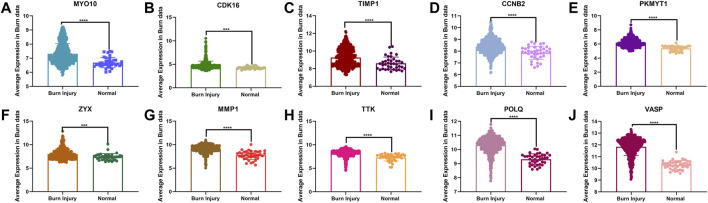
** (A–J)** Comparison of the expression levels of 10 hub genes between normal and burn injury groups.

**FIGURE 8:**
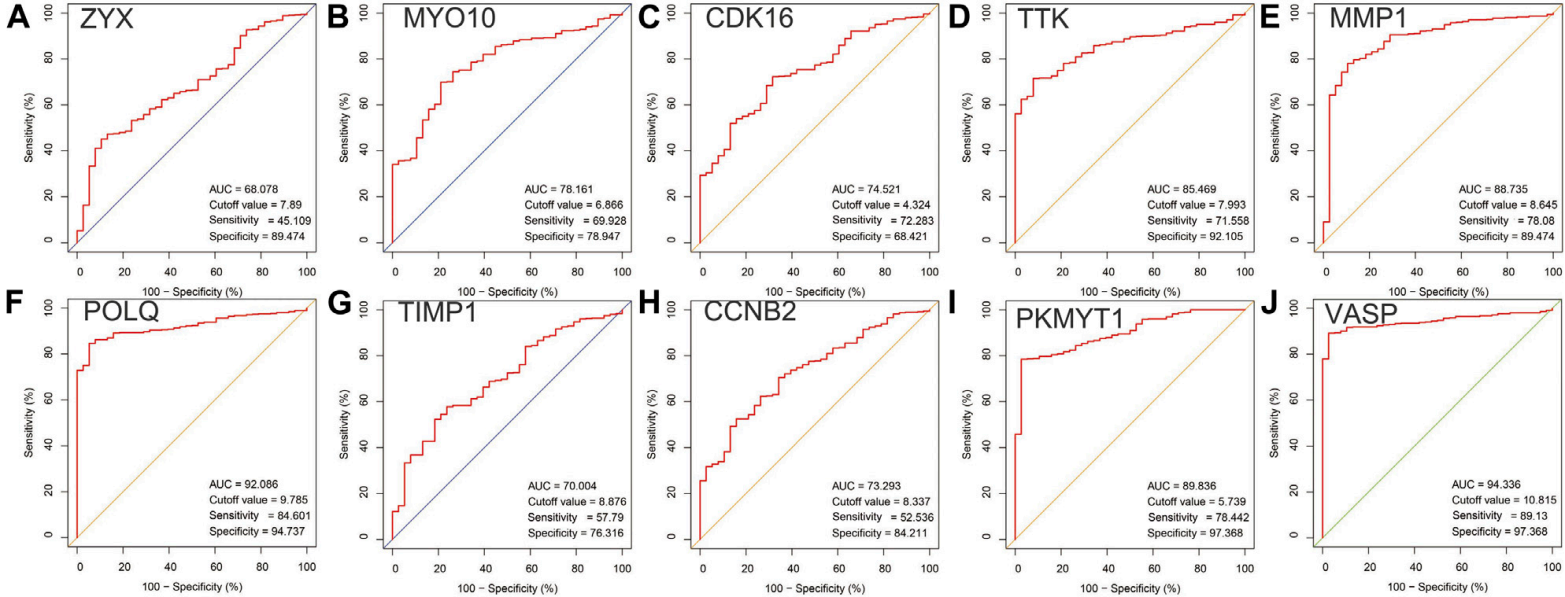
** (A–J)** Receiver operating characteristic curves of 10 hub genes in PPI network.

In the analysis of the expression level of hub genes, we found that burn tissues were significantly upregulated compared with normal tissues. Among them, CDK16 and ZYX were upregulated with *p* < 0.001, CCNB2, TTK, POLQ, MYO10, MMP1, TIMP1, VASP, and PKMYT1 with *p* < 0.0001 (Figure 7). The high expression of hub genes may be involved in the progression and basic pathological process of burns. In addition, the ROC curves were constructed based on the acquired sensitivity and specificity, thus we can judge the diagnostic ability of each gene. The AUC of all hub genes reached 0.68 (Figure 8).

### Infiltrating Immune Cells and Histologic Scoring in Burn Injury

**FIGURE 9:**
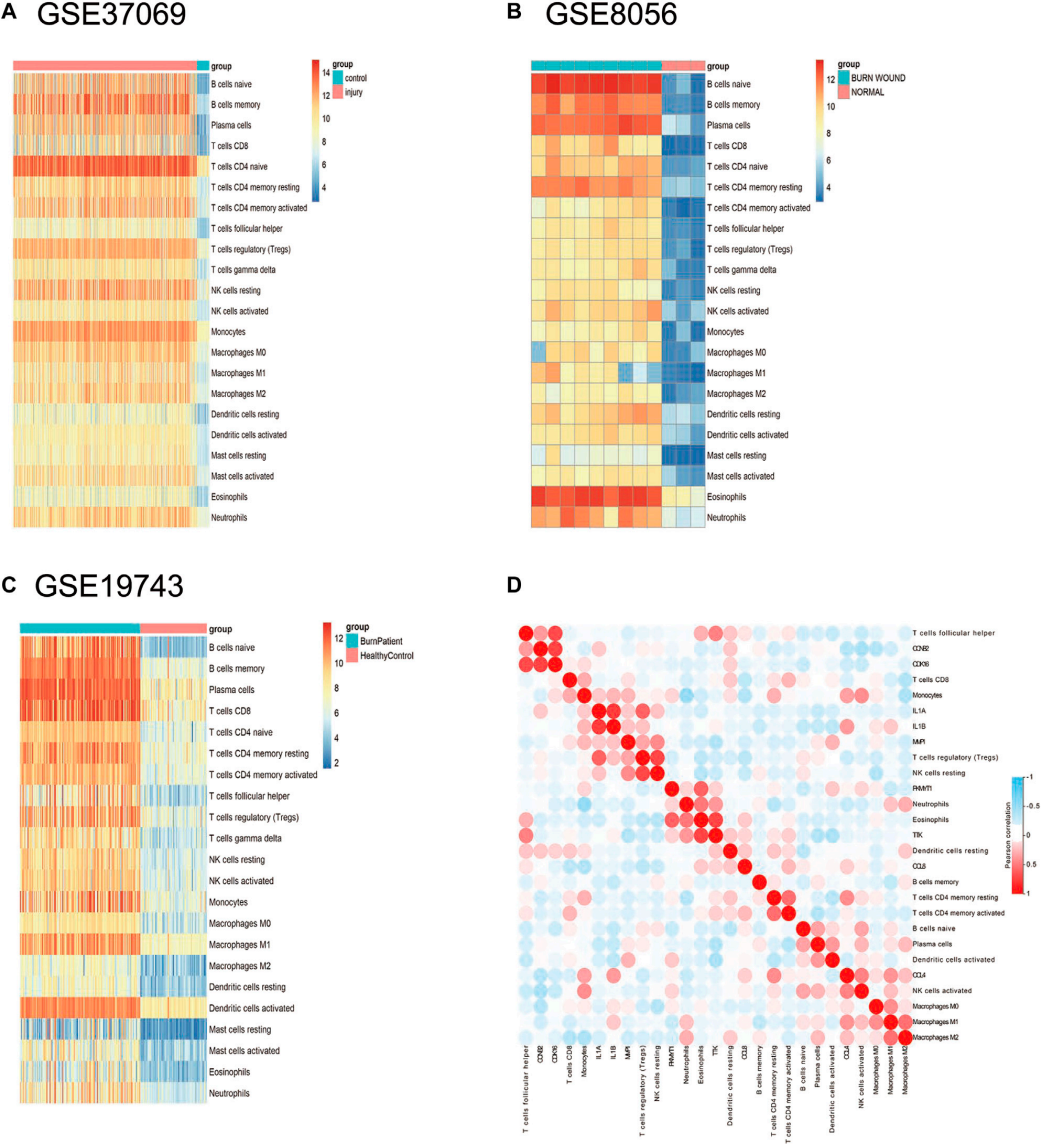
Immune infiltration analysis in the three datasets. **(A)** The expression heatmap of 22 immune cells in GSE37069; **(B)** the expression heatmap of 22 immune cells in GSE8056; **(C)** the expression heatmap of 22 immune cells in GSE19743; **(D)** correlation heatmap of immune cells in burn injury.

**FIGURE 10:**
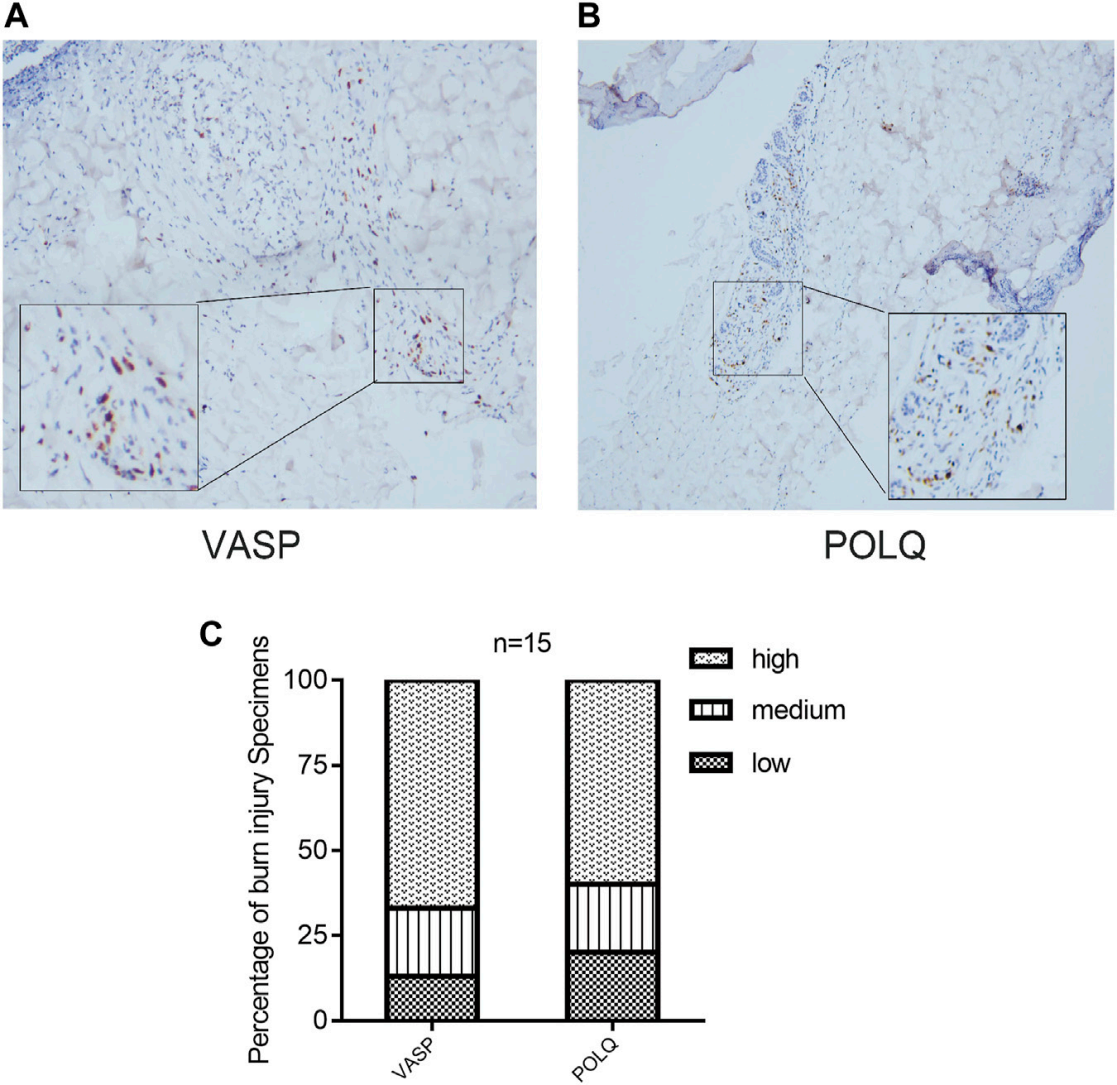
Immunohistochemistry staining and histologic scoring. IHC staining demonstrated that VASP **(A)** and POLQ **(B)** showed high expression; **(C)** results of histologic scoring and analysis.

Based on the results of the immune infiltration analysis on the GSE37069, GSE8056, and GSE19743 datasets, we constructed a heatmap of immune cell expression for each dataset (Figures 9A–C). In addition, we constructed the correlation heatmap for visualizing the correlation of infiltrating immune cells and some hub genes (Figure 9D). Compared with normal tissues, burn injury tissues have a high expression of a variety of immune cells, which indicates that burn-injured tissues have a complex immune infiltration in the pathological process, and multiple cells work together to form, develop, and repair burn injury through the inflammatory process. Since all 10 hub genes have good diagnostic values, we selected two genes with AUC >0.9 for further validation. Finally, we evaluated the expression of VASP and POLQ, the IHC staining results demonstrated that VASP and POLQ were highly expressed in the burn injury tissues (Figure 10).

## Discussion

Burn injury is common in daily life and may affect anyone, it is characterized by high morbidity and mortality (Jeschke et al., 2020). The main characteristics of burns are abnormal activation of the immune function, uncontrolled inflammation, metabolic changes, and infections (Sood et al., 2016; Stanojcic et al., 2018). These challenges may be unpredictable and may lead to organ failure and sepsis in patients (Church et al., 2006; Tejiram et al., 2019). Burn injuries require prompt treatment at the very early stages to reduce the possibility of irreversible consequences. However, the key molecules and functional alterations leading to this process are largely unknown; therefore, the identification of early biomarkers is needed to inhibit the worsening of the inflammatory response and promote wound healing in the early stage. We conducted a comprehensive bioinformatic analysis on a cohort of 676 burn patients and 103 healthy controls and identified 84 DEGs in the burn injury group (32 upregulated and 52 downregulated). The results of the enrichment analysis and GSEA showed that these genes are closely related to immune response, inflammation, stress, and immune cell activation. In addition, the top 10 burn-related genes identified in the PPI network showed similar functional characteristics and diagnostic values for burn injuries.

First, we identified 84 DEGs in the three GEO datasets. Biological process enrichment was performed and revealed that it was primarily enriched in the metabolic process, regulation of cell death, regulation of immune response, regulation of defense responses, regulation of leukocyte activation, and regulation of other innate immune responses in the burn injury. Biological responses, such as innate immune responses and metabolic processes, have been identified to play a vital role in burn injury (Lord et al., 2014; Williams and Herndon, 2017). Moreover, the upregulated DEGs were enriched in the cell cycle and neutrophil degranulation. Studies have reported that neutrophil degranulation plays a central role in the early stages of burn injury. To prevent endotoxemia and systematic inflammation response, the neutrophils are recruited to the site of inflammation by a mass of cytokines, and they kill pathogens through phagocytosis and degranulation (Walsh et al., 2000; Yu and Sun, 2020). The downregulated genes were primarily enriched in the regulation of cellular responses to stress, regulation of kinase activity, and regulation of transferase activity. This process is closely associated with the breakdown of proteins and a highly catabolic state after burn injury (Wise et al., 2019), thus the pathological basis of burns may be caused by a variety of potential pathways. Various factors, including genomics, oxidative stress, and immune dysfunction, play an important role in its progression, which indicates that immune inhibitors may be an important treatment for burn injury.

We identified 10 hub genes from the PPI network (CCNB2, MYO10, TTK, POLQ, VASP, TIMP1, CDK16, MMP1, ZYX, and PKMYT1), which are associated with the underlying pathological mechanism of burn injury and can be regarded as important target genes; future research should focus on these genes and explore their role in burn injury. However, the mechanisms underlying burn injury are largely unclear. To clarify the potential functions of burn-related genes, we performed GSEA of the hub genes. We found that some pathways are activated in burn injury, such as the VEGF signaling pathway, vascular smooth muscle contraction, complement and coagulation cascades, B cell receptor signaling pathway, and T cell receptor signaling. Most burn injuries usually present vascular destruction, and studies have shown that VEGFA/VEGFR2 signal transduction pathway may be involved in vascular reconstruction (Duchesne et al., 2019); at this time, the increase of proteins related to angiogenesis is more conducive to vascular remodeling (Demirci et al., 2015). Inflammation is another problem that cannot be ignored after a burn injury. Usually, in trauma, the interaction of the complement and coagulation systems provides the first line of defense against pathogens entering the body. Studies have shown that activation of the complement cascade is related to thrombosis and multiple organ failure. Meanwhile, it is often believed that there is an interaction between the coagulation system and complement system in sepsis (Oikonomopoulou et al., 2012), especially in severe burn injury cases.

Burn injuries are accompanied by immune and inflammatory responses. Burn injury not only cause damage to the skin tissue, but also cause immune system disorders due to the lack of barriers (Boldeanu et al., 2020), and immune dysfunction after burn injury leads to disordered signaling pathways in various cells. Burn injury can trigger early and severe pro-inflammatory CD4^+^ T cell responses in the immune system, suggesting that the injury may be a signal of CD4^+^ T cell activation. In addition, it also includes the upregulation of pro-inflammatory cytokines (TNF-α, interleukin-1β, and IL-6) and other processes (Purcell et al., 2006; Abbas et al., 2018). Of note, progesterone mediated oocyte metabolism was also observed in GSEA, a series of endocrine reactions occur after burn injury, in which almost all known hormones are involved (Ballian et al., 2010). Using CIBERSORT, we explored the correlation of burn injury and 22 infiltration immune cell types and we found that 22 immune-infiltration cells were significantly changed during the injury process. The immune infiltration level can be drastically altered during this process, especially in B cells and T cells. We found that there is an upregulation of eosinophils and naive B cells in GSE8056, which is consistent with previous research that reported that elderly patients have eosinophil infiltration in the early stage of burn wound healing (Ringnér, 2008). Identifying the changes in immune cells is important for clarifying the mechanism of wound formation and development in burn injuries.

Moreover, in the present study, we analyzed the diagnostic utility of 10 hub genes, and the ROC revealed that most of the genes showed promising diagnostic value. The AUC curves of the nine hub genes were reached 0.70. In particular, vasodilator-stimulated phosphoprotein (VASP) and DNA polymerase theta (POLQ) both showed excellent discriminative power in detecting burn injury in skin tissue. However, the relationship between VASP and POLQ and its mechanism in burn injury has not yet been investigated. VASP is a cytoskeletal effector protein that plays an important role in immunity and is related to motility, adhesion, and sensory capacity in many cells (Kwiatkowski et al., 2003). POLQ is a central modulator in the repair of double-strand breaks from external pressure (Higgins et al., 2010), although the physiological functions associated with this protein are not yet fully understood, we speculate that this protein may be involved in the complex cellular stress process of burn injury. Based on the above considerations, these genes might play an important role in burn injury and have the potential to be used as diagnostic biomarkers in the future. Therefore, it is necessary to conduct further analyses to evaluate the effects of these genes.

Although this study is the first to explore the pathogenesis of burn injury by integrated bioinformatics analysis, it still presents some limitations and shortcomings. The data in the database are still relatively limited and incomplete. Due to the lack of clinical information, we could not perform further stratified analysis of patients based on relevant clinical characteristics. Future research should focus on additional *in vivo* and *in vitro* experiments to clarify the role of hub genes and their underlying mechanisms.

In conclusion, this study explored the underlying molecular mechanisms of burn injury by comprehensive bioinformatic analysis, and we also identified 10 hub genes with excellent diagnostic value through enrichment analysis, PPI network and ROC curves, this study is crucial for elucidating biological mechanisms and exploring related molecular targets in burn injury.

## Data Availability

This study analyzed three publicly available datasets, which can be found in the Gene Expression Omnibus (GEO) database.This data can be found here: GSE8056, GSE19743, and GSE37069.

## Footnotes

1
http://www.ncbi.nlm.nih.gov/geo/.

2
http://metascape.org.

3
http://string-db.org.

4
http://cibersort.stanford.edu/.
